# Combination therapy versus monotherapy: retrospective analysis of antibiotic treatment of enterococcal endocarditis

**DOI:** 10.1186/s12879-025-10451-2

**Published:** 2025-01-20

**Authors:** Razan Saman, Christopher P. Primus, Robert West, Simon J. Woldman, Jonathan A. T. Sandoe

**Affiliations:** 1 Medical Microbiology, Leeds Teaching Hospitals Trust, Leeds, UK; 2 Cardiology, Barts Heart Centre, St Bartholomew’s Hospital, Barts Health NHS Trust, London, UK; 3 Leeds Institute of Health Sciences, University of Leeds, Leeds, UK; 4 Healthcare Associated Infection Group, Leeds Institute of Medical Research, University of Leeds, Leeds, UK; 5 Imperial College Healthcare NHS Trust, Du Cane Road, W12 0HS London, UK

**Keywords:** *Enterococcus faecalis*, Infective endocarditis, Biofilm, Gentamicin, Amoxicillin

## Abstract

**Background:**

Guidelines suggest treating fully penicillin-susceptible *Enterococcus faecalis* strains causing infective endocarditis with amoxicillin combined with gentamicin or ceftriaxone, but clinical evidence to support this practice is limited and monotherapy cohorts were excluded from studies. We describe antibiotic treatment, complications, and outcomes in patients with *Enterococcus faecalis* infective endocarditis, specifically comparing monotherapy versus combination therapy.

**Methods:**

Retrospective analysis of prospectively collected cohort of patients with definite or possible infective endocarditis from 2 English centres between 2006 and 2021. The primary outcome was 30-day mortality. Secondary outcomes included acute kidney injury, relapse, and clinical cure.

**Results:**

178 individuals were included: median age was 72 years (interquartile range 60–79), male sex majority (138, 78%) and mostly native valve endocarditis (108, 61%). Thirty-nine patients (22%) received monotherapy (penicillin/glycopeptide/linezolid/daptomycin), 128 (72%) combination with gentamicin, 11 (6%) combination with ceftriaxone. Patients on combination therapy with gentamicin had a statistically significant lower 30-day mortality than those treated with monotherapy (21 (16.4%) versus 15 (38.5%) *p* = 0.035) and higher rates of clinical cure (101 (78.9%) versus 23 (59.0%) *p* = 0.018). Patient receiving gentamicin were more likely to experience acute kidney injury (64 (50%) versus 11 (28.2%) *p* = 0.057). Ceftriaxone combination was associated with poor outcomes, but the sample size was small.

**Conclusion:**

Patients treated with combination gentamicin therapy had better clinical outcomes than patients treated with monotherapy. Low-dose gentamicin regimens were associated with acute kidney injury. Patients treated with combinations were different to those treated with monotherapy and confounding remains a concern with observational analyses. An adequately powered clinical trial is needed to determine optimal treatment of enterococcal endocarditis.

**Clinical trial number:**

Not applicable.

**Supplementary Information:**

The online version contains supplementary material available at 10.1186/s12879-025-10451-2.

## Introduction

The majority of cases of infective endocarditis (IE) are caused by bacteria, and *Enterococcus* species account for about 10–15% of all cases [1, 2]. *Enterococcus faecalis* is the most frequently isolated species, accounting for 90% of cases of enterococcal endocarditis [3]. Current treatment requires prolonged courses of antibiotics, often combined with surgery, but despite this, the long-term prognosis remains poor with mortality ranging from 11–35% [4].

International IE guidelines from the European Society of Cardiology (ESC), American Heart Association (AHA) and British Society for Antimicrobial Chemotherapy (BSAC) suggest fully penicillin-susceptible strains are treated with amoxicillin combined with gentamicin but the clinical evidence base to support this practice is limited [5–7]. There is concern about the potential nephrotoxicity of aminoglycosides, particularly as many of the patients suffering with *Enterococcus faecalis* infective endocarditis (EFIE) are advanced in age [8]. Furthermore, high-level aminoglycoside resistance (HLAR) has been reported in 43% of *Enterococcus faecalis*, which has driven a search for antibiotic alternatives to aminoglycosides [9].

Several observational cohort studies have suggested that ampicillin plus ceftriaxone may be as effective as ampicillin plus gentamicin for non-HLAR EFIE and that this regimen appears to be safe and associated with less nephrotoxicity [10–12]. However, to date there are no adequately powered randomised controlled trials have confirmed the benefit of adding either gentamicin or ceftriaxone to a cell-wall acting agent. Additionally, these few existing observational studies excluded patients treated with monotherapy, introducing bias into the analysis of effectiveness.

The aim of this study was to describe antibiotic treatment, renal complications, and outcomes in all patients with EFIE, with particular reference to patients treated with monotherapy versus combination therapy.

## Methods

### Study design

A retrospective study of prospectively collected cases was designed with consideration of, and is reported according to, STROBE criteria [13].

### Setting

Two separate UK tertiary cardiothoracic centres: Leeds Teaching Hospitals NHS Trust (LTHT) and Barts Heart Centre, part of Barts Health NHS Trust (BHC) were involved. All consecutive EFIE coded episodes occurring between 2006 and 2018 from LTHT and between 2015 and 2021 from BHC were included. Demographic, clinical, treatment, and outcome data were obtained from institutional clinical databases and patient medical records and inputted in a database created specifically for the purposes of the study (Microsoft Excel, 2010).

### Participants

The study population included adult patients (≥ 18 years of age) treated for a definite or possible diagnosis of any type of IE and *Enterococcus faecalis* from blood, brain abscess pus, splenic pus, major arterial embolus, heart valve tissue/prosthesis or CIED (e.g., pacemaker lead). Enterococci were identified by Gram stain appearance, colony morphology and matrix assisted laser desorption time of flight mass spectroscopy (MALDI TOFF/MS) or polymerase chain reaction (PCR) analysis. Susceptibility testing was carried out according to the European Committee on Antimicrobial Susceptibility Testing (EUCAST) methodology [14]. Patients meeting the above inclusion criteria but treated with a suboptimal regimen with palliative intent were excluded. This study was conducted with approval from the respective institution’s Caldicott guardians, and informed consent was not required from the patients.

### Variables

IE was defined as definite or possible according to the modified Duke criteria [15]. The Charlson comorbidity index (CCI) was used at admission to measure overall comorbidity [16]. A patient’s first episode of IE was defined as the episode diagnosed in the period of the study, and subsequent episodes were assessed to confirm if they were relapses or recurrences [17].

Appropriate antimicrobial agents were administered as per ESC/BSAC guidelines in consultation with a medical microbiologist. They included benzylpenicillin, amoxicillin, ceftriaxone, or substitutions with alternative active agents (e.g., vancomycin, teicoplanin, daptomycin) [5, 17]. The dose of antibiotic was considered appropriate when directed to treat EFIE by the treating clinician, again taking local guidelines and adjustments for renal function into account. Surgery included any operation intervention for endocarditis treatment.

The primary outcome was clinical cure; defined as the absence of relapse or death within the 6 months following completion of antibiotic therapy. Adverse effects recorded were acute kidney injury (AKI) and toxicity (pancytopenia, vestibular toxicity). AKI was defined as a sudden increase (≤ 48 h) in serum creatinine of ≥ 0.3 mg/dL or an increase of ≥ 50% over baseline creatinine during a 7-day period at any point after treatment [18]. Treatment failure was defined as no apparent clinical improvement (i.e., persistence of fever > 38^o^C or persistently positive blood cultures) after 10 days of targeted antibiotic treatment. Relapse was defined as IE or metastatic complication caused by *Enterococcus faecalis* occurring within 6 months of the initial episode and reinfection was defined as a second episode of IE caused by *E. faecalis*, 6 months or more after the first episode [19].

### Statistical analysis

Patient characteristics were cross tabulated with treatment type. Age was categorised in order to make analysis easier to interpret and account for nonlinearity of effect. Comparisons were then facilitated by Pearson’s chi-square test. Both univariable and multivariable logistic regressions were undertaken for regression of 30-day mortality and also for subsequent AKI on patient characteristics including the variable of interest, namely treatment type. Statistically significant associations were identified at the 5% level and the final multivariable models were selected to have only significant terms. All statistical analysis was undertaken in the R statistical software environment R version 4.2.3 [20].

## Results

### Participants

178 episodes of definite or possible EFIE were included, 112 from LTHT and 66 from BHC. We excluded 4 patients who were less than 18 years old, 26 who had an alternative diagnosis, and 1 who was treated with a suboptimal regimen for palliative intent.

### Descriptive data

Baseline demographic and clinical characteristics of patients receiving monotherapy versus combination therapy are shown in Table 1. Overall, the median age was 72 years (interquartile range (IQR) 60–79) with a male sex majority (138, 78%) and a median CCI of 4 (IQR 2–7). Most patients had native valve IE (108, 61%), whilst prosthetic valve IE accounted for 35% (*n* = 62) of our cohort the majority of which were late prosthetic valve IE (52,29%).(See supplementary data Table 1) The populations managed at the two centres differed significantly; patients from LTHT were older (median age 75 (IQR, 63–93)), had more co-morbidities (particularly chronic kidney disease (CKD) and cancer), and a higher frequency of penicillin allergy than BHC. A significant proportion had moderate or severe comorbidity at presentation particularly at LTHT (median CCI, (IQR) overall, 4(2–7); LTHT, 5(3–7); BHC 2(1–6)). Patients attending BHC had more intracardiac and extracardiac complications (disseminated infection) and a greater proportion of them underwent surgery (53% vs. 22%, *p* < 0.0001). In total 60 patients underwent surgery at a median 18 days (IQR,8–36) from positive culture.

**Table 1:** Comparison of characteristics and outcomes of 178 patients with *Enterococcus faecalis* infective endocarditis grouped by antibiotic therapy regimen

	Monotherapy *N* = 39	Combined gentamicin *N* = 128	Other combination *N* = 11	*p*-value
Age (%)				
24–65	5 ( 12.8)	46 ( 35.9)	5 ( 45.5)	0.063
65–75	14 ( 35.9)	29 ( 22.7)	2 ( 18.2)	
75–93	20 ( 51.3)	53 ( 41.4)	4 ( 36.4)	
Hospital (%)				
BHC	4 ( 10.3)	52 ( 40.6)	10 ( 90.9)	< 0.001
LTHT	35 ( 89.7)	76 ( 59.4)	1 ( 9.1)	
Sex (%)				
Female	11 ( 28.2)	28 ( 21.9)	1 ( 9.1)	0.388
Male	28 ( 71.8)	100 ( 78.1)	10 ( 90.9)	
CCI (%)				
0–2	7 ( 17.9)	47 ( 37.0)	3 ( 27.3)	0.149
3–6	18 ( 46.2)	53 ( 41.7)	4 ( 36.4)	
≥7	14 ( 35.9)	27 ( 21.3)	4 ( 36.4)	
Intravenous drug use (%)				
No	38 ( 97.4)	116 ( 90.6)	10 ( 90.9)	0.379
Yes	1 ( 2.6)	12 ( 9.4)	1 ( 9.1)	
Diabetes mellitus (%)				
Absent	32 ( 82.1)	98 ( 77.2)	6 ( 54.5)	0.159
Present	7 ( 17.9)	29 ( 22.8)	5 ( 45.5)	
CKD(%)				
Absent	19 ( 48.7)	93 ( 73.2)	5 ( 45.5)	0.006
Present	20 ( 51.3)	34 ( 26.8)	6 ( 54.5)	
Haemodialysis (%)				
No	33 ( 84.6)	125 ( 97.7)	11 (100.0)	0.004
Yes	6 ( 15.4)	3 ( 2.3)	0 ( 0.0)	
AKI on presentation (%)				
Absent	21 ( 53.8)	88 ( 68.8)	3 ( 27.3)	0.01
Present	18 ( 46.2)	40 ( 31.2)	8 ( 72.7)	
Duke (%)				
Definite	28 ( 71.8)	113 ( 88.3)	9 ( 81.8)	0.045
Possible	11 ( 28.2)	15 ( 11.7)	2 ( 18.2)	
Gentamicin sensitivity*** (%)				
Sensitive	10 ( 25.6)	76 (59.4 )	0 (0 )	< 0.001
Resistant	14 (35.9 )	6 (46.9)	3 (27.3 )	
Valve involvement				
Aortic valve (%)				
No	21 ( 53.8)	63 ( 49.2)	5 ( 45.5)	0.838
Yes	18 ( 46.2)	65 ( 50.8)	6 ( 54.5)	
Mitral valve (%)				
No	32 ( 82.1)	100 ( 78.1)	8 ( 72.7)	0.771
Yes	7 ( 17.9)	28 ( 21.9)	3 ( 27.3)	
Tricuspid valve (%)				
No	35 ( 89.7)	124 ( 96.9)	10 ( 90.9)	0.168
Yes	4 ( 10.3)	4 ( 3.1)	1 ( 9.1)	
Pulmonary valve (%)				
No	39 (100.0)	127 ( 99.2)	11 (100.0)	0.822
Yes	0 ( 0.0)	1 ( 0.8)	0 ( 0.0)	
Multiple valves affected (%)				
No	35 ( 89.7)	106 ( 82.8)	10 ( 90.9)	0.484
Yes	4 ( 10.3)	22 ( 17.2)	1 ( 9.1)	
NVE (%)				
No	16 ( 41.0)	50 ( 39.1)	4 ( 36.4)	0.955
Yes	23 ( 59.0)	78 ( 60.9)	7 ( 63.6)	
Early PVE (%)				
No	34 ( 87.2)	123 ( 96.1)	11 (100.0)	0.075
Yes	5 ( 12.8)	5 ( 3.9)	0 ( 0.0)	
Late PVE$ (%)				
No	33 ( 84.6)	86 ( 67.2)	7 ( 63.6)	0.096
Yes	6 ( 15.4)	42 ( 32.8)	4 ( 36.4)	
CIED IE (%)				
No	36 ( 92.3)	123 ( 96.1)	11 (100.0)	0.461
Yes	3 ( 7.7)	5 ( 3.9)	0 ( 0.0)	
Surgery (%)				
No	31 ( 81.6)	83 ( 64.8)	3 ( 27.3)	0.003
Yes	7 ( 18.4)	45 ( 35.2)	8 ( 72.7)	
Outcomes characteristics				
AKI on treatment (%)				
No	28 ( 71.8)	64 ( 50.0)	4 ( 36.4)	0.028
Yes	11 ( 28.2)	64 ( 50.0)	7 ( 63.6)	
30-day mortality (%)				
No	24 ( 61.5)	107 ( 83.6)	7 ( 63.6)	0.008
Yes	15 ( 38.5)	21 ( 16.4)	4 ( 36.4)	
Length of stay (median [IQR])	46 [29, 69]	43 [32, 53.7]	49 [17.5, 57.5]	0.454
Clinical cure (%)				
No	16 ( 41.0)	27 ( 21.1)	5 ( 45.5)	0.018
Yes	23 ( 59.0)	101 ( 78.9)	6 ( 54.5)	

Abbreviations: IQR interquartile range, AKI acute kidney injury, CIED-IE cardiac implantable electronic devices infective endocarditis, NVE native valve endocarditis, PVE prosthetic valve endocarditis, CKD chronic kidney disease, CCI Charlson comorbidity index

$Prosthetic valve IE occurring > 1 year after implantation of valve

***Sensitivity data available for 109 patients LTHT *n* = 94 BHC *n* = 15

Patients were commenced on 5 main targeted antibiotic regimen categories: amoxicillin monotherapy (18 patients, 10%), glycopeptide monotherapy (19 patients, 11%), penicillin (amoxicillin/ benzylpenicillin) and gentamicin (116 patients, 65%), glycopeptide and gentamicin (11 patients, 6%) amoxicillin and ceftriaxone (9 patients 5%). Other antibiotic regimens included daptomycin monotherapy (1 patient, 1%), amoxicillin and vancomycin (2 patient, 1%), linezolid monotherapy (1 patient, 1%), linezolid and gentamicin (1 patient, 1%).

Amoxicillin was administered intravenously at 2 g 4-hourly. Vancomycin was administered intravenously with an initial loading dose according to creatinine clearance and weight, followed by a maintenance regimen with trough levels of 15 to 20 mg/L. Teicoplanin was used intravenously (*n* = 10, 6%), mainly for outpatient parenteral antibiotic therapy (OPAT) purposes to facilitate discharge, at 12 mg/kg and adjusted to pre-dose levels of 30–40 mg/L. Daptomycin was used intravenously at doses of 6 mg/kg once daily. Linezolid was used intravenously and orally at 600 mg every 12 h. Streptomycin was administered at a dose of 7.5 mg/kg body weight every 12 h to maintain pre-dose levels ≤ 3 mg/kg. Gentamicin was given intravenously at with low dose regimens of either 1 mg/kg 12-hourly or 3 mg/kg once daily then adjusted to pre-dose levels of < 1 mg/l and 1-hour post-dose levels of 3–5 mg/l. Dose adjustments were made according to renal function when required.

The median duration of overall targeted treatment was 41 days (IQR, 28–44 days). This includes the 72 patients that had a switch in their antibiotics (Indication: OPAT regimen *n* = 11; AKI *n* = 20; toxicity *n* = 3; treatment failure *n* = 6; superadded infection *n* = 4).

**Fig. 1:**
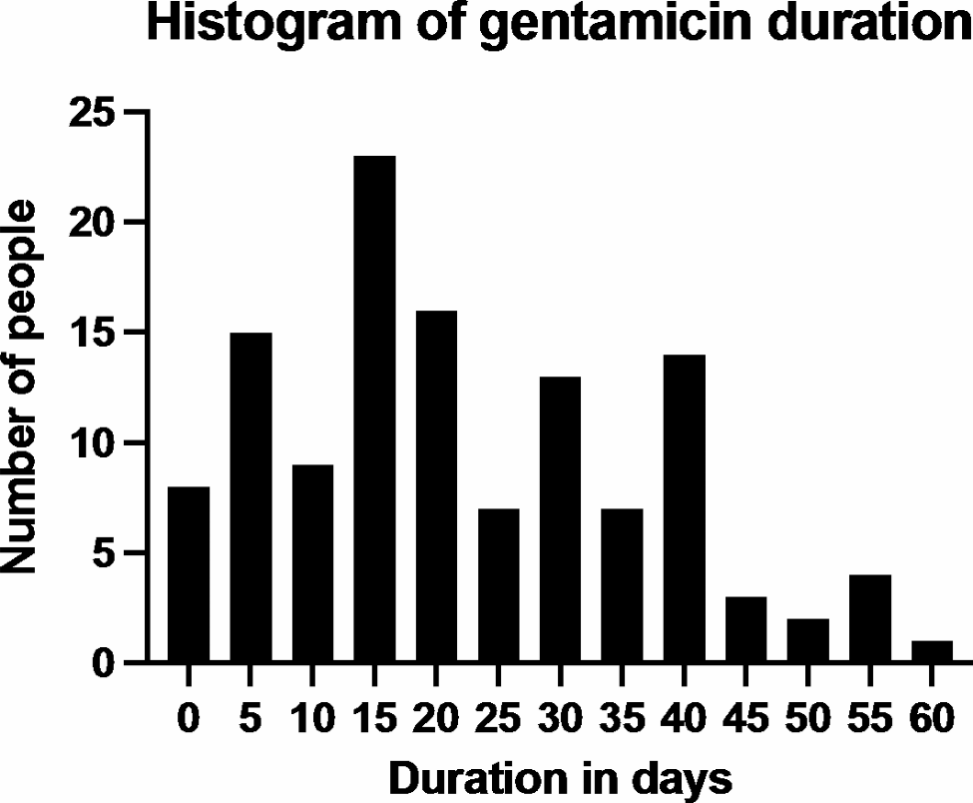
Duration of gentamicin therapy in 128 out of 178 episodes of *Enterococcus faecalis* infective endocarditis

Details of the duration of gentamicin therapy are shown in Fig. 1, with median duration 18 days (IQR, 12–34 days). Three of 178 (1.7%) of the enterococci isolate were penicillin resistant and Of 178 patients only 109 (61%) had aminoglycoside susceptibility data available (LTHT *n* = 94 and BHC *n* = 15); 25 (23%) of the *Enterococcus faecalis* strains showed HLAR. Of the 128 patients with known gentamicin duration, 36 patients had gentamicin stopped within 14 days because of: AKI, 11 (31%); HLAR, 4 (11%); reason not documented, 21 (58%).

### Outcome data

The median age was 76 years (IQR, 68–82 years) for patients receiving monotherapy and 70 years (IQR, 59–79 years) for those receiving a combination therapy (either ceftriaxone or gentamicin) with patients receiving monotherapy having a higher CCI 6 (IQR, 3–8) than other combination regimens. These patients treated with monotherapy also had lower rates of clinical cure and higher rates of 30-day mortality as demonstrated in Tables 1 and 2.

**Table 2:** Outcomes of 178 episodes of EFIE treated with monotherapy versus combination therapy

Variable	Monotherapy (any) (*N* = 39)	Combination (any) (*N* = 139)	Combination therapy with gentamicin (*N* = 128)	Combination therapy with ceftriaxone (*N* = 9)	Combination therapy other (*n* = 2)	*P*-value (significant < 0.005)
Median age, years (IQR)	76 (68–82)	70 (59–79)	71 (58.3–79)	71 (60–80)	41.5 (40–43)	0.0461
Male sex, n (%)	28 (72%)	110 (79%)	100 (78%)	8 (89%)	2 (100%)	0.703
CCI, median (IQR)	6 (3–8)	4 (1.75-6)	4 (2–6)	4 (2–9)	2 (0–4)	0.038
Outcome						
Clinical cure	23 (56%)	107 (77%)	101 (79%)	4 (44%)	2 (100%)	0.02
Treatment failure requiring change of antimicrobials, n (% in treatment group)	3 (8%)	4 (3%)	3 (2%)	1(11%)	0	0.361
30-day mortality, n (% in treatment group)	8 (21%)	25 (18%)	21 (16%)	4 (44%)	0	0.287
One-year mortality, n (% in treatment group)	16 (41%)	40 (29%)	35 (27%)	5 (56%)	0	0.171
Relapse n (% in treatment group)	1 (3%)	0	0	0	0	0.128

The multivariable analysis excluded patients treated with combinations other than gentamicin as numbers were too small to yield useful output. Multivariable analysis demonstrated older patients, those with AKI at presentation and those treated with monotherapy had significantly higher 30-day mortality (Table 3). Patients treated with gentamicin combination therapy, older patients, those with AKI at presentation, those who had surgery and those managed in BHC were more likely to experience AKI during the course of their treatment (Table 4).

**Table 3:** Multivariable logistic regression analysis of risk factors for 30-day mortality in 167 patients with *Enterococcus faecalis* endocarditis

	Survived	Died	OR (univariable)	OR (multivariable)
Age				
24–65	51 (91.1)	5 (8.9)	-	-
65–75	38 (84.4)	7 (15.6)	1.88 (0.56–6.78, *p* = 0.312)	0.85 (0.19 to 3.62, *p* = 0.826)
75–93	49 (63.6)	28 (36.4)	5.83 (2.24–18.25, *p* = 0.001)	3.88 (1.36 to 12.96, *p* = 0.017)
Sex				
Female	30 (75.0)	10 (25.0)	-	-
Male	108 (78.3)	30 (21.7)	0.83 (0.37 − 0.97, *p* = 0.664)	-
Hospital				
BHC	57 (86.4)	9 (13.6)	-	-
LTHT	81 (72.3)	31 (27.7)	2.42 (1.11–5.76, *p* = 0.033)	3.05 (1.05 to 10.29, *p* = 0.051)
CCI				
0–2	50 (87.7)	7 (12.3)	-	-
3–6	60 (80.0)	15 (20.0)	1.79 (0.70–4.99, *p* = 0.243)	-
7 or more	27 (60.0)	18 (40.0)	4.76 (1.83–13.59, *p* = 0.002)	-
NVE				
No	50 (71.4)	20 (28.6)	-	-
Yes	88 (81.5)	20 (18.5)	0.57 (0.28–1.16, *p* = 0.119)	-
Valvular endocarditis				
No	9 (64.3)	5 (35.7)	-	-
Yes	129 (78.7)	35 (21.3)	0.49 (0.16–1.67, *p* = 0.224)	-
AKI at presentation				
No	95 (84.8)	17 (15.2)	-	-
Yes	43 (65.2)	23 (34.8)	2.99 (1.46 to 6.24, *p* = 0.003)	2.55 (1.08 to 6.17, *p* = 0.034)
Duke’s Criteria				
Definite	114 (76.0)	36 (24.0)	-	-
Possible	24 (85.7)	4 (14.3)	0.53 (0.15 to 1.48, *p* = 0.265)	0.24 (0.06 to 0.79, *p* = 0.030)
Surgery event				
No	85 (72.6)	32 (27.4)	-	-
Yes	53 (88.3)	7 (11.7)	0.35 (0.13 to 0.81, *p* = 0.021)	-
Antibiotic therapy				
Combined gentamicin	107 (83.6)	21 (16.4)	-	-
Monotherapy	24 (61.5)	15 (38.5)	3.18 (1.43 to 7.08, *p* = 0.004)	2.78 (1.08 to 7.34, *p* = 0.035)

**Table 4:** Multivariable logistic regression analysis of risk factors for acute kidney Injury in 167 patients with *Enterococcus faecalis* endocarditis

	No AKI	AKI on treatment	OR (univariable)	OR (multivariable)
Age				
24–65	34 (60.7)	22 (39.3)	-	-
65–75	17 (37.8)	28 (62.2)	2.55 (1.15–5.79, *p* = 0.023)	3.47 (1.22–10.47, *p* = 0.023)
75–93	45 (58.4)	32 (41.6)	1.10 (0.55–2.23, *p* = 0.792)	2.17 (0.80–6.24, *p* = 0.135)
Sex				
Female	26 (65.0)	14 (35.0)	-	-
Male	70 (50.7)	68 (49.3)	1.80 (0.88–3.83, *p* = 0.113)	-
AKI at presentation				
No	72 (64.3)	40 (35.7)	-	-
Yes	24 (36.4)	42 (63.6)	3.15 (1.69-6.00, *p* < 0.001)	3.60 (1.60–8.50, *p* = 0.002)
Hospital				
BHC	17 (25.8)	49 (74.2)	-	-
LTHT	79 (70.5)	33 (29.5)	0.14 (0.07–0.28, *p* < 0.001)	0.19 (0.08–0.42, *p* < 0.001)
CCI				
0–3	29 (50.9)	28 (49.1)	-	-
3–6	29 (55.8)	23 (44.2)	0.82 (0.38–1.75, *p* = 0.609)	-
6–15	38 (55.9)	30 (44.1)	0.82 (0.40–1.66, *p* = 0.576)	
NVE				
Absent	39 (55.7)	31 (44.3)	-	-
Present	57 (52.8)	51 (47.2)	1.13 (0.62–2.07, *p* = 0.701)	-
Valvular endocarditis				
No	8 (57.1)	6 (42.9)	-	-
Yes	88 (53.7)	76 (46.3)	1.15 (0.38–3.64, *p* = 0.802)	-
Duke criteria				
Definite	75 (50.0)	75 (50.0)	-	-
Possible	21 (75.0)	7 (25.0)	0.33 (0.12–0.80, *p* = 0.018)	-
Surgery event				
No	77 (65.8)	40 (34.2)	-	-
Yes	19 (31.7)	41 (68.3)	4.15 (2.16–8.21, *p* < 0.001)	4.61 (1.91–11.75, *p* = 0.001)
Antibiotic therapy				
Combined gentamicin	64 (50.0)	64 (50.0)	-	-
Monotherapy	28 (71.8)	11 (28.2)	0.39 (0.17–0.84, *p* = 0.019)	0.37 (0.13- 1.00, *p* = 0.057)

## Discussion

The aim of this study was to describe antibiotic treatment, complications, and outcomes in all patients with EFIE, particularly to explore the outcomes of patients treated with monotherapy versus combination therapy. The rationale cited in guidelines for use of combination therapy to treat EFIE is to overcome a described phenomenon of ‘tolerance’ exhibited by some enterococci to certain cell-wall agents [6]. Improved bactericidal activity was first observed in vitro and in vivo with gentamicin combination and then in experimental animal trials for ceftriaxone combinations [21].

Overall, monotherapy was found to be used in 22% of episodes, surgery was required in 34% and AKI on treatment occurred in 46% of patients. Gentamicin was the most frequently used agent in combination, with a small number of patients treated with ceftriaxone (*n* = 9) and other combinations. Combination therapy with gentamicin was associated with lower mortality, but an increased risk of AKI, compared to monotherapy. Although low dose gentamicin is used in IE to reduce the risk of AKI, this analysis suggests it still poses a risk of toxicity. Ceftriaxone combination was associated with poor outcomes despite the majority of these patients (7, 78%) undergoing surgery.

The biggest EFIE study to date in Spain examined the treatment of 291 patients with definite or possible IE: no patients were treated with monotherapy but rates of surgery (40%), AKI (46%) in patients treated with gentamicin and ampicillin were very similar to those seen in this study, 35% and 50%, respectively [10]. It compared outcomes in patients treated with ampicillin plus gentamicin to those in patients treated with ampicillin plus ceftriaxone and found no significant differences in mortality [10]. This is contrary to our finding of higher 30-day mortality in patients on amoxicillin plus ceftriaxone, albeit in small numbers of patients. Another retrospective Spanish cohort study of 69 patients compared patients treated with ampicillin in combination with either ceftriaxone or gentamicin and found no difference in health outcomes but renal failure was again more common in the aminoglycoside group [1]. This study did not include patients treated with monotherapy. A systematic review examining evidence to support ampicillin and ceftriaxone for enterococcal endocarditis found it was safe, had similar outcomes to ampicillin and gentamicin but the studies were “inadequately designed or powered” [22].

Relapse was uncommon (< 1%) in our study, the single case being treated with monotherapy (see supplementary data Table 1). A recent multicentre retrospective study in France looked at the rate and clinical features of relapses, and investigated whether they were impacted by choice of the antibiotic regimen [23]. They found no impact on the risk of relapses (relapse definition of 1 year as opposed to 6 months in our study) between ceftriaxone or gentamicin as combined therapy. Only 3% of their cohort was treated with monotherapy compared to 22% in this study. They concluded, despite their small cohort that amoxicillin monotherapy should not be used in this indication due to high risk of relapse. We also found that patients on monotherapy had higher 30-day mortality and lower rates of clinical cure, however, patients treated with monotherapy were different from those treated with gentamicin, having higher rates of CKD, haemodialysis and AKI on admission. Only a small proportion (9, 5%) of our patients were treated with ceftriaxone combinations, mainly at BHC. Patients from BHC were more likely to have surgery as part of their management and had more extracardiac and intracardiac complications as a result of their EFIE. This may contribute to the differences in AKI and mortality when comparing BHC to LTHT. Patients with AKI at presentation were less likely to receive combination therapy rendering renal function a prominent factor for influencing choice of therapy. It is noteworthy that a retrospective study (*N* = 71) of follow-on treatment with teicoplanin monotherapy, after initial standard therapy, found no difference in relapse rates or mortality [24].

Multivariable analyses were carried out using prespecified variables based on prior literature and the research questions and limited due to the small sample size. Stepwise analysis using all available variables may have yielded different results, but we feel this approach is less valid. The multivariable analysis of factors affecting mortality gave plausible results that were largely as expected: mortality increased with increased age and was not affected by valve type (found previously for EFIE). Lower mortality with Duke possible cases is also expected and may reflect earlier detection of IE or less severe disease. Reduced mortality with surgery is as expected. A poorer prognosis with AKI at presentation is plausible. The lack of a significant effect on mortality with increasing Charlson comorbidity index is unexpected, highlighting the great potential for confounding in this type of retrospective observational study.

A significant number of patients were treated with gentamicin before susceptibility testing for HLAR was determined. This resulted in these patients being treated with gentamicin without the likely benefit whilst still being exposed to the risks of gentamicin ototoxicity and nephrotoxicity. Consideration should be given to delaying the start of gentamicin therapy until the sensitivity results are known.

We recruited from 2 large geographically separate sites to improve generalisability, nonetheless, we only included 2 sites, both heart centres, and our patients are likely not to be representative of all EFIE patients. It is important to note that the setup of the 2 tertiary cardiothoracic centres in our cohort was different and this would have affected the services they offered. LTHT and BHC both provide hub and spoke models accepting patients to their centres who require surgical intervention, with larger numbers of patients being treated in BHC. Furthermore, in BHC local guidelines are based on ESC [5] favouring the use of ceftriaxone combination therapy, whereas in LTHT BSAC guidelines [7] are the standard.

Our study has the expected limitations of a retrospective observational study including missing data, and loss to follow up (usually due to transfer to another hospital precluding data collection), this may introduce bias in the analysis. Treatment regimens were not determined by randomisation but by local decision making (by a medical microbiologist or infectious diseases physician) and reasons for monotherapy use were not collected. The prolonged period of time required to collect data on a rare condition mean the changes in clinical guidelines and practice over time are an additional confounder that cannot be easily adjusted for [5, 7]. Referral bias may have led to inclusion of more severely ill patients and those more likely to need for surgery. However, our patients were older and predominantly male, in keeping with other enterococcal endocarditis epidemiological studies [25]. Another notable limitation in our study was the small number of patients that made up the ceftriaxone combination group.

## Conclusion

In conclusion, in our cohort of EFIE patients, combination therapy with gentamicin was associated with lower 30-day mortality but higher rates of AKI. Monotherapy was used in a significant proportion of patients with low relapse rates. Confounding affects all such observational studies and an appropriately designed randomised trial is needed to determine the benefits of both gentamicin and ceftriaxone over monotherapy.

## Electronic supplementary material

Below is the link to the electronic supplementary material.

Supplementary Material 1

## Data Availability

The datasets used and/or analysed during the current study are available from the corresponding author on reasonable request.

## Abbreviations

AHA: American Heart Association
AKI: Acute kidney injury
BHC: Barts Heart Centre
BSAC: British Society for Antimicrobial Chemotherapy
CCI: Charlson comorbidity index
CIED: Cardiac Implantable Electronic Devices
CKD: Chronic kidney disease
EFIE: Enterococcus faecalis infective endocarditis
ESC: European Society of Cardiology
HLAR: High-level aminoglycoside resistance
IE: Infective endocarditis
IQR: Interquartile range
LTHT: Leeds Teaching Hospitals NHS Trust
OPAT: Outpatient antimicrobial therapy
