# Supplementary material for: Combination therapy versus monotherapy: retrospective analysis of antibiotic treatment of enterococcal endocarditis
Source: BMC Infect Dis. 2025 Jan 20;25:92. doi: 10.1186/s12879-025-10451-2 (PMC11748874; doi:10.1186/s12879-025-10451-2)
Supplement: Supplementary file 1 — Supplementary Material 1 [file 12879_2025_10451_MOESM1_ESM.docx]

**Supplementary data**

**Table 1: Comparison of characteristics and outcomes of 178 patients with *Enterococcus faecalis* infective endocarditis grouped by centre**

| **Variable** | **All patient (n=178)** | **LTHT (n=112)** | **BHT (n=66)** | **P-value** |
| --- | --- | --- | --- | --- |
| **Patient Characteristics** | | | | |
| Median age in years, y, (IQR) | 72 (60-79.3) | 75 (63-93) | 68 (58-76.3) | 0.013 |
| Male sex n (%) | 138 (78%) | 83 (74%) | 55 (83%) | 0.194 |
| **Comorbidities** | | | | |
| Median Charlson Co-morbidity Index, (IQR) | 4 (2-7) | 5 (3-7) | 2 (1-6) | 0.001 |
| Intravenous recreational drug use n (%) | 14 (8%) | 7 (6%) | 7 (11%) | 0.388 |
| Diabetes mellitus n (%) | 41 (23%) | 21 (19%) | 20 (30%) | 0.097 |
| Chronic kidney disease n (%) | 60 (34%) | 44 (39%) | 16 (24%) | 0.049 |
| Haemodialysis n (%) | 9 (5%) | 8 (7%) | 1 (2%) | 0.157 |
| AKI on presentation n (%) | 66 (37%) | 37 (33%) | 29 (44%) | 0.152 |
| Cancer n (%) | 36 (21%) | 17 (15%) | 19 (29%) | 0.035 |
| COPD n (%) | 30 (17%) | 16 (14%) | 14 (21%) | 0.300 |
| Penicillin allergy n (%) | 19 (11%) | 16 (14%) | 3 (5%) | 0.047 |
| **Type of IE n (%)** | | | | |
| Native valve n (%) | 108 (61%) | 67 (60%) | 41 (62%) | 0.874 |
| Prosthetic valve n (%) | 62 (35%) | 37 (33%) | 25 (38%) | 0.520 |
| Early prosthetic valve n (%) | 10 (6%) | 10 (9%) | 0 (0%) | 0.014 |
| Late prosthetic valve n (%) | 52 (29%) | 27 (24%) | 25 (38%) | 0.061 |
| ICED-IE n (%) | 10 (6%) | 7 (6%) | 3 (5%) | 0.747 |
| **Valve involvement** | | | | >0.05 |
| Aortic alone n (%) | 89 (50%) | 62 (55%) | 27 (41%) | 0.088  0.184  >0.999  >0.999  0.019  0.259 |
| Mitral alone n (%) | 37 (21%) | 20 (18%) | 18 (27%) |  |
| Tricuspid alone n (%) | 9 (5%) | 6 (5%) | 3 (5%) |  |
| Pulmonary alone n (%) | 1 (1%) | 1 (1%) | 0 (0%) |  |
| More than one affected valve n (%) | 28 (16%) | 12 (11%) | 16 (24%) |  |
| Unknown(/other) n (%) | 14 (8%) | 11 (10%) | 3 (5%) |  |
| **Treatment Characteristics** | | | | |
| Duration of antibiotic treatment day median, (IQR) | 41 (28-44) | 41 (28-44) | 42 (31.8-47.3) | 0.038 |
| HLAR susceptibility (n=109) *** n (%) | 23 (21%) | 21 (22%) | 2 (13%) | 0.513 |
| **Outcome characteristics** | | | | |
| Extracardiac foci of infection* n (%) | 76 (43%) | 28 (25%) | 48 (73%) | <0.0001 |
| Intracardiac complications** n (%) | 43 (24%) | 13 (12%) | 30 (45%) | <0.0001 |
| Surgery n (%) | 60 (34%) | 25 (22%) | 35 (53%) | <0.0001 |
| Median length of stay, IQR | 44.5 (30.8-60) | 45 (32.3-62.8) | 43.5 (26.5-54.3) | 0.161 |
| 30-day mortality n (%) | 35 (20%) | 27 (25%) | 8 (12%) | 0.034 |
| Clinical cure n (%) | 130(73%) | 75 (67%) | 55 (83%) | 0.023 |
| Abbreviations: IQR interquartile range, AKI acute kidney injury, ICED-IE Implantable cardiac electronic devices infective endocarditis  $Prosthetic valve IE occurring >1 year after implantation of valve.  *Extracardiac foci of infection were defined as septic and/or embolic events outside the endocardium  **Intracardiac complications were defined as abscess, pseudoaneurysm, fistula, enlarging vegetation, leaflet perforation/rupture,  ***Sensitivity data available for 109 patients LTHT n=94 BHT n=15 | | | | |
